# Intra-abdominal aortic graft infection: prognostic factors associated with in-hospital mortality

**DOI:** 10.1186/1471-2334-14-215

**Published:** 2014-04-22

**Authors:** Matthias Garot, Pierre-Yves Delannoy, Agnès Meybeck, Béatrice Sarraz-Bournet, PierVito d’Elia, Thibaud d’Escrivan, Patrick Devos, Olivier Leroy

**Affiliations:** 1 Service de Réanimation Médicale et Maladies Infectieuses, Hôpital Chatiliez, Tourcoing 59, France; 2 Service de chirurgie vasculaire, Hôpital Chatiliez, Tourcoing 59, France; 3 Département d’anesthésie, Hôpital Chatiliez, Tourcoing 59, France; 4 Département de bio statistiques, CHRU, Lille 59, France; 5 Service de Réanimation Médicale et Maladies Infectieuses, Hôpital Chatiliez, Rue du Président Coty, Tourcoing 59208, France

**Keywords:** Aortic graft, Aortic surgery, Prosthetic infection, Prognosis

## Abstract

**Background:**

Mortality associated with aortic graft infection is considerable. The gold standard for surgical treatment remains explantation of the graft. However, prognostic factors associated with early mortality due to this surgical procedure are not well-known.

**Methods:**

Retrospective analysis of patients admitted in our center between January 2006 and October 2011 for aortic graft infection. The primary endpoint was in-hospital mortality. A bivariate analysis of characteristics of patients associated with in-hospital outcome was performed.

**Results:**

Twenty five evaluable patients were studied. All patients were male. Their mean age was 67 ± 8.4 years. Most of them (92%) had severe underlying diseases. An *in situ* prosthetic graft replacement, mainly using cryopreserved arterial allografts, was performed in all patients, excepted one who underwent extra-anatomic bypass. Causative organisms were identified in 23 patients (92%). The in-hospital mortality rate was 48%. Among pre-operative characteristics, age ≥ 70 years, creatinine ≥ 12 mg/L and C reactive protein ≥ 50 mg/L were significantly associated with in-hospital mortality. Hospital mortality rates increased with the number of risk factor present on ICU admission, and were 0%, 14.3%, 85.7% and 100% for 0, 1, 2 and 3 factors, respectively. The only intra-operative factor associated with prognosis was an associated intestinal procedure due to aorto-enteric fistula. SAPS II, SOFA score and occurrence of medical or surgical complications were postoperative characteristics associated with in-hospital mortality.

**Conclusion:**

Morbidity and mortality associated with surgical approach of aortic graft infections are considerable. Age and values of creatinine and C Reactive protein on hospital admission appear as the most important determinant of in hospital mortality. They could be taken into account for guiding the surgical strategy.

## Background

Infection represents one of the major complications associated with prosthetic aortic graft with a reported incidence ranging 0.5% to 2% [1]. It be could lower as demonstrated by Vogel et al. [2]. They studied 12,626 patients who underwent, between 1987 and 2005, open repair for abdominal aortic aneurysms. The 2-year rate of aortic graft infection was 0.19%. The occurrence of bacteremia or surgical site infection during the index hospitalization was significantly associated with the development of aortic graft infection.

The optimal management of aortic graft infection remains unclear and many questions on diagnosis, surgical management, and antibiotic therapy are unresolved [3]. For example, if the gold standard for surgical treatment is always graft explantation, the best techniques for reperfusion are unknown. The extra-anatomic bypassing technique using an axillofemoral graft was long considered the technique associated with the lower re-infection rate. It now appears than *in situ* revascularisation procedure could be more effective [4,5]. Nevertheless, uncertainty remains about the best graft for *in situ* replacement. The choice between normal graft, silver-coated polyester graft, rifampin-impregnated graft, allografts or autogenous vein could be difficult [3,4].

Aortic graft infection is associated with considerable mortality and morbidity. In the Vogel et al. study, in-hospital mortality rate for patients readmitted for infection was 18% [2]. However, recent studies reported higher mortality rates [6]. To the best of our knowledge only a few studies reported factors associated with prognosis of patients suffering from aortic graft infection. We created in 2005 a multidisciplinary group including vascular surgeons, microbiologists, infectious diseases physicians, anesthesiologists and intensivists to optimize the management of patients with prosthetic vascular graft infection. This group has been managing a growing number of patients from public and private hospitals of the Nord-Pas de Calais area (4 millions of inhabitants), in the north of France. We reported in 2012 our experience about the first 85 evaluable patients treated in our center between 2005 and 2009 [7]. Given the lack of prognostic data about in-hospital outcome of patients suffering from aortic graft infection, our goal was to identify preoperative, intraoperative and postoperative characteristics of patients associated with poor outcome.

## Methods

### Study population

All patients admitted from January 2006 to October 2011 in the Intensive Care Unit (ICU), and the infectious diseases and surgical vascular departments of Tourcoing Hospital with a diagnosis of intra abdominal aortic graft infection were included in this retrospective study. In accordance with French law, approval of an Ethics Committee was not required for a cohort study that did not modify existing diagnosis or therapeutic strategies.

All patients had a previous aortofemoral or aortoiliac bypass. They were considered to suffer from definite intra abdominal aortic graft infection if at least two of the three following criteria were present: (a) clinical signs of infection either systemic (fever, chills, septic shock) or in the area of the prosthesis (i.e., enteric aortic fistula, intra-operative gross purulence, failure of graft incorporation), (b) biological signs of infection (C-reactive protein > 10 mg/l, white blood count > 10,000/mm^3^) or radiological signs of infection (pathognomonic perigraft air or fluid, abscess) and (c) positive culture of intraoperative specimens or blood cultures (for potentially ‘contaminant’ pathogens such as coagulase-negative *Staphylococcus, P. acnes* or corynebacteria at least two intraoperative specimens or blood cultures or, at least one intraoperative specimen and one blood culture are required) [8].

### Surgical procedures and antimicrobial management

All patients underwent a surgical procedure including complete debridement of devitalized and infected tissues around the prosthesis, total graft excision and *in situ* reconstruction or extra-anatomic bypass grafting.

Blood specimens for culture were drawn from all febrile patients (over 38.5°C). Bacterial samples were collected intraoperatively. Multiple intraoperative samples were cultured on blood agar plates with standard aerobic and anaerobic methods. Antibiotic susceptibility patterns were interpreted in accordance with recommendations of the Comité de l’Antibiogramme de la Société Française de Microbiologie [9].

Empirical broad-spectrum intravenous antibiotic therapy was started immediately after intra-operative sampling and was secondarily adapted to microbiological results. In case of severe sepsis or septic shock, empirical therapy was started before surgical management, immediately after blood samples had been collected. Initial antimicrobial treatment was considered appropriate when all causative pathogens were *in vitro* susceptible to at least one of the antibiotics in the regimen. The duration of intravenous antimicrobial treatment was at least 6 weeks.

### Data collection and definitions

On hospital admission, data about demographic characteristics, underlying diseases, prior vascular procedures, and biological findings were recorded.

Diabetes mellitus and chronic obstructive pulmonary diseases (COPD) were defined according to criteria proposed by the Expert Committee on the diagnosis and classification of diabetes mellitus and the American Thoracic Society, respectively [10,11]. A body mass index (BMI) ≥ 30 Kg/m^2^ defined obesity [12]. Immunosuppression included transplant, cancer, acquired immunodeficiency syndrome, immunosuppressive drugs and other immunodeficiency conditions. Risk of the surgical procedure was evaluated by the POSSUM score [13]. A classification of the preoperative physical status (ASA score) of the American Society of Anaesthesiologist was used for anaesthetic risk prediction [14].

Data about surgical management and intra-operative care were collected. They included type and duration of surgery, duration of aortic clamping, volume of intra-operative fluid administration and blood transfusion, and use of vasopressors.

On ICU admission, the severity of illness was assessed by the Simplified Acute Physiology score II (SAPS II), and the Sequential Organ Failure Assessment (SOFA) score [15,16].

During ICU stay, we recorded data about the use and duration of mechanical ventilation, renal replacement therapy, vasopressors, and occurrence of complications. Septic shock, acute respiratory distress syndrome, acute renal failure or hospital acquired infections were defined according to usual criteria and categorized as medical complications. Vascular complications such as graft thrombosis, bleeding, or distal embolization were categorized as surgical complications.

### Outcome assessment

The primary outcome was the in-hospital mortality rate, defined as any death, regardless of its cause, occurring within the hospitalization in our center.

### Statistical analysis

Descriptive analyses were performed to check and resume data. Quantitative variables are reported as means ± SD. Qualitative variables are reported as number and percentage. Continuous variables were compared using Student’s *t* test. When appropriate, continuous variables were analyzed as categorical variables using cut-points determined by method of box plots. Categorical variables were compared using the Chi-square test or Fisher’s exact test when the Chi-square was not appropriate. Differences between groups were considered significant for variables yielding a p value ≤ 0.05. No multivariate analysis was performed.

All statistical analyses were performed using SAS Software, V9.1.

## Results

During the study period, 26 patients suffering from intra-abdominal aortic graft infection were admitted in our institution. One patient was not included due to missing data.

Main characteristics of patients on hospital admission are summarized in Table 1. All patients were male and most of them (96%) had severe underlying diseases such as hypertension, coronary artery disease, COPD or obesity. Infection occurred within the 4 months following initial aortic graft surgery in 7 cases (28%). Four of the 25 patients were admitted in ICU before surgical management.

**Table 1:** Preoperative characteristics of patients

**Variables**	
Age (years)	67 ± 8.4
Comorbidities	
Obesity (BMI > 30 kg/m^2^)	11 (44%)
Hypertension	18 (72%)
Coronary artery disease	16 (64%)
Diabetes mellitus	4 (16%)
COPD	7 (28%)
Immunosuppression	6 (24%)
ASA Score ≥ 3	21 (84%)
POSSUM score	46.1 ± 10.7
Preoperative severe sepsis	7 (28%)
Previous graft	
Aortounifemoral bypass graft/Dacron	5 (20%)/5
Aortobifemoral bypass graft/Dacron	14 (56%)/13
Aortobiiliac bypass/Dacron	6 (24%)/6
Biological data	
Creatinine (mg/L)	15.0 ± 13.8
C Reactive Protein (mg/L)	69.1 ± 76.1

Data are expressed as mean ± standard deviation values or numbers (%) of patients.

Eight patients exhibiting on admission an anastomotic rupture underwent an emergency surgical procedure, one of whom died during surgery. The other 17 patients had elective surgery. An *in situ* prosthetic graft replacement was performed in all patients, excepted one who underwent an extra-anatomic bypass. Implanted aortic grafts were wrapped with omental flaps in 21 patients. Cryopreserved arterial allografts were mainly used (n = 22/23). In 4 patients, with aorto-enteric fistula, an intestinal procedure was required. Durations of operation and aortic clamping, blood transfusions, colloid and crystalloid administration volumes and use of vasopressors are summarized in Table 2.

**Table 2:** Intra-operative characteristics of patients

**Variables**	
Mean duration of operation (minutes)	270 ± 81
Mean duration of aortic clamping (minutes)	118 ± 50
Suprarenal aortic clamping	6 (24%)
Associated intestinal procedure	4 (16%)
Blood transfusion	
Packed red cells (number of patients/units)	25 (100%)/5.7 ± 2.1
Fresh frozen plasma (number of patients/units)	21 (84%)/3.2 ± 1.8
Platelets (number of patients)	4 (16%)
Volume of intra-operative colloid administration (ml)	1020 ± 549
Volume of intra-operative crystalloid administration (ml)	3740 ± 1280
Use of vasopressor(s)	20 (80%)

Data are expressed as mean ± standard deviation values or numbers (%) of patients.

Causative organisms of aortic graft infection were identified in 23 patients (92%). Preoperative blood cultures were positive in 13 cases. In the remaining 10 cases, only bacterial cultures from intra-operative samples were positive. Thirty causative pathogens were isolated. In 8 patients, more than one pathogen was identified. Organisms were Gram positive cocci (methicillin susceptible *S. aureus* n = 3, methicillin resistant *S. aureus* n = 2, coagulase negative staphylococcus n = 3, *Streptococcus* spp. n = 3, *Enterococcus* spp. n = 2), Gram negative bacilli (*E. coli* n = 8; enterobacteriaceae n = 2), Gram positive bacilli (n = 3), anaerobes (n = 2) and *Candida* spp. (n = 2).

Antimicrobial therapy was begun before surgical procedures in 17 cases (68%) because either preoperative identification of causative organism(s) (n = 10) or preoperative presence of severe sepsis (n = 7). Most antimicrobial regimens included a wide spectrum betalactam (piperacillin-tazobactam n = 12, imipenem n = 4, 3rd generation cephalosporin n = 3) combined with an agent effective against methicillin-resistant *staphylococcus* spp. (glycopeptide n = 16, daptomycin n = 3, linezolid n = 1). Initial antimicrobial therapy was considered adequate in 22 patients (96%).

After surgical procedures, 24 patients were admitted in the ICU. Main data about severity scores, vasopressors use, mechanical ventilation, and biological characteristics on ICU admission are reported Table 3. During ICU stay, 16 patients (67%) developed medical complications, acute renal failure (n = 13), septic shock (n = 11), hospital-acquired infections (n = 5) and acute respiratory distress syndrome (ARDS) (n = 2). Renal replacement therapy was required for 12 (50%) patients. The numbers of days without mechanical ventilation, vasopressors and/or renal replacement therapy on D28 for patients requiring such treatments are reported Table 3. Surgical complications occurred in 15 patients (63%). Most frequent complications were bleeding (n = 8), graft thrombosis (n = 5), distal embolization (n = 4) and aortic graft-enteric fistula (n = 1). Eight patients (33%) underwent a secondary surgical procedure.

**Table 3:** Characteristics of patients on ICU admission and during ICU stay

**Variables**	
On ICU admission	
SAPS II	47 ± 16
SOFA Score	8 ± 4
Mechanical ventilation	23 (92%)
Use of vasopressor(s)	13 (54%)
Biological data	
Creatinine (mg/L)	14.1 ± 11.6
Lactate (mmol/L)	3.0 ± 2.5
C Reactive Protein (mg/L)	123 ± 84
PaO^2^/FiO^2^ (mmHg)	315 ± 155
During ICU stay	
Occurrence of medical complications	16 (67%)
Occurrence of surgical complications	15 (63%)
Number of days without mechanical ventilation at D28*	10.3 ± 10.6
Number of days without use of vasopressors at D28*	15.5 ± 11.3
Number of days without renal replacement therapy at D28*	7.3 ± 9.9

Data are expressed as mean ± standard deviation values or numbers (%) of patients. *Determined in patients treated with mechanical ventilation, vasopressors and/or renal replacement therapy.

Ten patients died in ICU. The mean length of ICU stay for non survivors was 13.2 ± 9.5 days. Fourteen patients were transferred from the ICU to medical or surgical wards, 14 ± 12 days after ICU admission. Among them, one patient died before hospital discharge. The in-hospital mortality rate was thus 48% (12/25). Comparison of pre-operative, intra-operative and post-operative patients’ characteristics between in-hospital survivors and non survivors is reported Table 4. Among pre-operative characteristics, age ≥ 70 years, creatinine ≥ 12 mg/L and C reactive protein ≥ 50 mg/L were significantly associated with in-hospital mortality. According to the number of these factors present on ICU admission, in hospital mortality rates were 0%, 14.3%, 85.7% and 100% for 0, 1, 2 and 3 factors, respectively. Interestingly, among the 12 patients < 70 years, only one patient died during hospital stay and he had pre-operative 13 mg/l creatinine and 235 mg/L C Reactive protein. Among the 13 patients, older than 70 years, 11 died during hospital stay. The 2 survivors had neither creatinine ≥ 12 mg/L nor C reactive protein ≥ 50 mg/L. Among intraoperative characteristics, only an associated intestinal procedure was associated with prognosis. Finally, among post-operative characteristics, non survivors had higher SAPS II and SOFA Score on ICU admission and exhibited more frequent medical and/or surgical complications.

**Table 4:** Bivariate analysis of factors associated with in-hospital death

**Variables**	**Survivors (n = 13)**	**Non survivors (n = 12)**	**p**
Pre-operative characteristics			
Age (years)	61.6 ± 6.5	74.5 ± 3.5	0.0006
Age ≥ 70 years	2	11	0.0002
Obesity (BMI > 30 kg/m^2^)	4	7	0.43
Hypertension	9	9	1
Coronary artery disease	9	7	0.69
Diabetes mellitus	3	1	0.6
COPD	5	2	0.39
Immunosuppression	3	3	1
ASA Score	2.9 ± 0.76	3.5 ± 0.67	0.09
Possum Score	42 ± 44	46 ± 55	0.06
Preoperative severe sepsis	2	5	0.20
Creatinine (mg/L)	10.4 ± 4.6	20.1 ± 18.4	0.03
Creatinine ≥ 12 mg/L	3	9	0.02
C Reactive Protein (mg/L)	16.0 ± 26.0	126.5 ± 202.0	0.006
C Reactive Protein ≥ 50 mg/L	2	8	0.008
Intra-operative characteristics			
Emergent surgical procedure for anastomotic rupture	2	6	0.1
Mean duration of operation (minutes)	273 ± 70	266 ± 95	0.70
Mean duration of aortic clamping (minutes)	103 ± 39	136 ± 57	0.15
Suprarenal aortic clamping	2	4	0.36
Associated intestinal procedure	0	4	0.04
Number of packed red cells units	5.3 ± 1.2	6.1 ± 2.8	0.51
Volume of intra-operative colloid administration (ml)	961 ± 518	1083 ± 596	0.61
Volume of intra-operative crystalloid administration (ml)	4003 ± 924	3454 ± 1571	0.33
Use of vasopressor(s)	9	11	0.32
Post-operative characteristics			
SAPS II on ICU admission	40.5 ± 8.2	54.7 ± 20.6	0.10
SOFA Score on ICU admission	6.5 ± 3.7	10.4 ± 3.9	0.05
Mechanical ventilation on ICU admission	12	11	1
Use of vasopressor(s) on ICU admission	5	8	0.12
Biological data on ICU admission			
Creatinine (mg/L)	11.2 ± 4.4	17.5 ± 16.2	0.25
Lactate (mmol/L)	2.1 ± 0.7	4.0 ± 3.5	0.10
C Reactive Protein (mg/L)	106.6 ± 74.0	142.3 ± 97.0	0.70
PaO^2^/FiO^2^ (mmHg)	365.3 ± 137.1	260.1 ± 160.7	0.10
Occurrence of medical complications during ICU stay	6	10	0.02
Occurrence of surgical complications during ICU stay	5	10	0.01
Renal replacement therapy during ICU stay	4	8	0.16

Data are expressed as mean ± standard deviation values or numbers (%) of patients.

One year after hospital discharge, all patients were still alive without recurrence of infection.

## Discussion

Twenty five patients suffering from intra-abdominal aortic graft infection were included in this retrospective study. All underwent graft excision. In-hospital morbidity and mortality were high. Prognostic analysis suggests that some characteristics of patients on hospital admission such as age and values of C-reactive protein and creatinine could predict in-hospital outcome.

In our series, postoperative complications occurred in 67% of patients and 48% of patients died during hospital stay. This mortality rate is in the high range on what is reported in the literature with mortality rates for aortic graft infection between 8 and 56.5% [17-27]. Most published studies focused on surgical aspect of aortic graft infection treatment but only few reported exhaustive data on surgical patients underlying conditions. Finally, search for predictive factors of perioperative mortality was not performed in most studies.

In 2004, Kieffer et al. reported a series of 179 consecutive surgery patients, from 1988 to 2002, and observed a 20.1% early postoperative mortality [22]. Batt among 82 consecutive aortic graft infection patients, from 2000 to 2008, found a 33% perioperative mortality rate [6]. The same author had reported in 2003 a lower perioperative death rate of 16.6% [21]. In our opinion, such discrepancies could only be explained by differences between patients undergoing surgical procedures, particularly underlying diseases. In the 2003 Batt’s study with 16.6% mortality, coronary artery disease, hypertension, diabetes mellitus, chronic obstructive pulmonary disease, were found in 36%, 41%, 14%, and 22% of patients, respectively [21]. In our series, the incidence of these underlying diseases was quite higher since it was 64%, 72%, 16%, and 28%, respectively and this might explain the differences in prognosis.

Prognostic factors associated with early postoperative mortality reported by Kieffer et al. were septic shock, presence of aorto-enteric fistula, emergency surgery, emergency allograft replacement, surgical or medical complications, and need for repeat surgery [22]. In our study we searched for pre, intra or post operative factors associated with in-hospital mortality. Among postoperative factors, occurrence of medical or surgical complications was associated with poor outcome, as in Kieffer’s study. SOFA score on ICU admission was also significantly higher in non survivors than in survivors. This score reflects organ failures occurring during surgery whereas septic shock reported by Kieffer fits only to hemodynamic failure. The single intra operative characteristic associated with prognosis was “associated intestinal procedure”. This is similar to the presence of aorto-enteric fistula previously reported. Surprisingly, durations of operation and aortic clamping, suprarenal clamping, volume of colloid or crystalloid administration, number of packed red cell units and vasopressors use were not statistically different in survivors and non survivors. Finally, we found that three preoperative factors, age ≥ 70 years, creatinine ≥ 12 mg/L, and C reactive protein ≥ 50 mg/L were associated with prognosis. The combination of these 3 prognostic factors seems interesting since in hospital mortality rate was 7.7% when no or only one factor was present and 91.6% when two or three factors were present. An age under 70 years could be a useful single factor to take in account as 91.6% of patients less than 70 years survived. Conversely, the survival rate of patients over 70 years was very low (15.4%). The survivors among the older patients had neither creatinine ≥ 12 mg/L nor C reactive protein ≥ 50 mg/L. In our mind these data could be considered before any surgical approach of an aortic graft infection. A surgical procedure in older patients with either creatinine ≥ 12 mg/L or C reactive protein ≥ 50 mg/L or both could be questionable. Long-term or lifelong suppressive antimicrobial therapy in patients who are not eligible for surgical device removal has been reported [28,29] with partial success. Medical treatment could appear as the best choice in patients for which graft excision is associated with no, or very low in-hospital survival rate.

Our study has numerous limits. First of all, it is retrospective and performed in a single centre. Although data collection was retrospective, it was exhaustive and only one patient was excluded for lack of data. Second, the number of included patients is low with only 25 studied patients. Nevertheless, the number of patients included in numerous other studies is similar [17,18,21,24,26]. Finally, the value of our statistical analysis is limited as no multivariate analysis was performed. As underlined by Peduzzi and Concato, logistic regression analysis needs between 10 and 15 events per co variable [30]. As we observed 12 events (in-hospital deaths) in this series, no multivariate analysis could be performed. Nevertheless, as data about prognostic factors are scarce, we believe our results and particularly the impact of preoperative factors such as age, and values of C reactive protein and creatinine add to the knowledge of this disease. Obviously, our results must be evaluated in a prospective and, ideally, multicenter study including more patients.

## Conclusion

In conclusion, morbidity and mortality associated with surgical approach of aortic graft infections are considerable. Age and values of creatinine and C Reactive protein on hospital admission appear as the most important determinant of in hospital mortality and, consequently, of surgical strategy.

## Abbreviations

ICU: Intensive care unit; COPD: Chronic obstructive pulmonary disease; BMI: Body mass index; MDRD: Modification of diet in renal disease; GFR: Glomerular filtration rate; POSSUM: Physiological and operative severity score for the enumeration of mortality; ASA: American Society of Anaesthesiology; SAPS: Simplified Acute Physiology Score; SOFA: Sepsis-related organ failure assessment; SD: Standard deviation.

## Competing interests

The authors declare that they have no competing interests.

## Authors’ contributions

MG collected data and helped to draft the manuscript, PYD participated in the design of the study, collected data and helped to draft the manuscript, AM collected data and helped to draft the manuscript, BSB collected data and helped to draft the manuscript, PVE collected data and helped to draft the manuscript, TE collected data and helped to draft the manuscript, PD performed the statistical analysis, and OL contributed to the design of the study and wrote the manuscript. All authors read and approved the final manuscript.

## Pre-publication history

The pre-publication history for this paper can be accessed here:

http://www.biomedcentral.com/1471-2334/14/215/prepub

## References

[B1] Seeger JM Management of patients with prosthetic vascular graft infection Am Surg 2000 14 166 177 10695748

[B2] Vogel TR Symons R Flum DR The incidence and factors associated with graft infection after aortic aneurysm repair J Vasc Surg 2008 14 264 269 10.1016/j.jvs.2007.10.030 18241747

[B3] FitzGerald SF Kelly C Humphreys H Diagnosis and treatment of prosthetic aortic graft infections: confusion and inconsistency in the absence of evidence or consensus J Antimicrob Chemother 2005 14 996 999 10.1093/jac/dki382 16269550

[B4] Pupka A Skora J Janczak D Plonek T Marczak J Szydełko T *In situ* revascularisation with silver-coated polyester prostheses and arterial homografts in patients with aortic graft infection–a prospective, comparative, single-centre study Eur J Vasc Endovasc Surg 2011 14 61 67 10.1016/j.ejvs.2010.10.005 21095143

[B5] O’Connor S Andrew P Batt M Becquemin JP A systematic review and meta-analysis of treatments for aortic graft infection J Vasc Surg 2006 14 38 45 10.1016/j.jvs.2006.02.053 16828424

[B6] Batt M Jean-Baptiste E O’Connor S Feugier P Haulon S Association Universitaire de Recherche en Chirurgie Vasculaire (AURC) Contemporary management of infrarenal aortic graft infection: early and late results in 82 patients Vascular 2012 14 129 137 10.1258/vasc.2011.oa0315 22661612

[B7] Legout L Sarraz-Bournet B D’Elia PV Devos P Pasquet A Caillaux M Wallet F Yazdanpanah Y Senneville E Haulon S Leroy O Characteristics and prognosis in patients with prosthetic vascular graft infection: a prospective observational cohort study Clin Microbiol Infect 2012 14 352 358 10.1111/j.1469-0691.2011.03618.x 21883666

[B8] Leroy O Meybeck A Sarraz-Bournet B D’Elia P Legout L Vascular graft infections Curr Opin Infect Dis 2012 14 154 158 10.1097/QCO.0b013e3283501853 22248976

[B9] Recommandations du Comité de l’Antibiogramme de la Société Française de Microbiologie http://www.sfm-microbiologie.org

[B10] Report of the Expert Committee on the Diagnosis and Classification of Diabetes Mellitus Diabetes Care 1997 14 7 1183 1197 9203460 10.2337/diacare.20.7.1183

[B11] American Thoracic Society Standards for the diagnosis and care of patients with chronic obstructive pulmonary disease Am J Respir Crit Care Med 1995 14 suppl 2 77 121 7582322

[B12] Garrow JS Webster J Quetelet’s index (W/H2) as a measure of fatness Int J Obes 1985 14 147 153 4030199

[B13] Copeland GP Jones D Walters M POSSUM: a scoring system for surgical audit Br J Surg 1991 14 356 360 10.1002/bjs.1800780327 2021856

[B14] American Society of Anesthesiology New classification of physical status Anesthesiol 1963 14 111 117

[B15] Le Gall JR Lemeshow S Saulnier F A new Simplified Acute Physiology Score (SAPS II) based on a European/North American multicenter study JAMA 1993 14 2957 2963 10.1001/jama.1993.03510240069035 8254858

[B16] Vincent JL Moreno R Takala J Willatts S De Mendonça A Bruining H Reinhart CK Suter PM Thijs LG The SOFA (Sepsis-related Organ Failure Assessment) score to describe organ dysfunction/failure Intensive Care Med 1996 14 707 710 10.1007/BF01709751 8844239

[B17] Young RM Cherry KJ Jr Davis PM Gloviczki P Bower TC Panneton JM Hallett JW Jr The results of *in situ* prosthetic replacement for infected aortic grafts Am J Surg 1999 14 136 140 10.1016/S0002-9610(99)00146-4 10487266

[B18] Lesèche G Castier Y Petit MD Bertrand P Kitzis M Mussot S Besnard M Cerceau O Long-term results of cryopreserved arterial allograft reconstruction in infected prosthetic grafts and mycotic aneurysms of the abdominal aorta J Vasc Surg 2001 14 616 622 10.1067/mva.2001.116107 11668314

[B19] Noel AA Gloviczki P Cherry KJ Jr Safi H Goldstone J Morasch MD Johansen KH United States Cryopreserved Aortic Allograft Registry Abdominal aortic reconstruction in infected fields: early results of the United States cryopreserved aortic allograft registry J Vasc Surg 2002 14 847 852 10.1067/mva.2002.123755 12021697

[B20] Chiesa R Astore D Frigerio S Garriboli L Piccolo G Castellano R Scalamogna M Odero A Pirrelli S Biasi G Mingazzini P Biglioli P Polvani G Guarino A Agrifoglio G Tori A Spina G Vascular prosthetic graft infection: epidemiology, bacteriology, pathogenesis and treatment Acta Chir Belg 2002 14 238 247 12244902 10.1080/00015458.2002.11679305

[B21] Batt M Magne JL Alric P Muzj A Ruotolo C Ljungstrom KG Garcia-Casas R Simms M *In situ* revascularization with silver-coated polyester grafts to treat aortic infection: early and midterm results J Vasc Surg 2003 14 983 989 10.1016/S0741-5214(03)00554-8 14603204

[B22] Kieffer E Gomes D Chiche L Fléron M-H Koskas F Bahnini A Allograft replacement for infrarenal aortic graft infection: early and late results in 179 patients J Vasc Surg 2004 14 1009 1017 10.1016/j.jvs.2003.12.040 15111853

[B23] Pirrelli S Arici V Bozzani A Odero A Aortic graft infections: treatment with arterial allograft Transplant Proc 2005 14 2694 2696 10.1016/j.transproceed.2005.06.098 16182787

[B24] Kitamura T Morota T Motomura N Ono M Shibata K Ueno K Kotsuka Y Takamoto S Management of infected grafts and aneurysms of the aorta Ann Vasc Surg 2005 14 335 342 10.1007/s10016-005-0006-4 15818454

[B25] Oderich GS Bower TC Cherry KJ Jr Panneton JM Sullivan TM Noel AA Carmo M Cha S Kalra M Gloviczki P Evolution from axillofemoral to *in situ* prosthetic reconstruction for the treatment of aortic graft infections at a single center J Vasc Surg 2006 14 1166 1174 10.1016/j.jvs.2006.02.040 16765233

[B26] Batt M Jean-Baptiste E O’Connor S Bouillanne PJ Haudebourg P Hassen-Khodja R Declemy S Farhad R In-situ revascularisation for patients with aortic graft infection: a single centre experience with silver coated polyester grafts Eur J Vasc Endovasc Surg 2008 14 182 188 10.1016/j.ejvs.2008.02.013 18440252

[B27] Saleem BR Meerwaldt R Tielliu IF Verhoeven EL van den Dungen JJ Zeebregts CJ Conservative treatment of vascular prosthetic graft infection is associated with high mortality Am J Surg 2010 14 47 52 10.1016/j.amjsurg.2009.05.018 20074700

[B28] Roy D Grove DI Efficacy of long-term antibiotic suppressive therapy in proven or suspected infected abdominal aortic grafts J Infect 2000 14 184 187 10.1016/S0163-4453(00)80014-6 10841097

[B29] Baddour LM Infectious Diseases Society of America’s Emerging Infections Network Long-term suppressive antimicrobial therapy for intravascular device-related infections Am J Med Sci 2001 14 209 212 10.1097/00000441-200110000-00011 11678518

[B30] Peduzzi P Concato J Kemper E Holford TR Feinstein AR A simulation study of the number of events per variable in logistic regression analysis J Clin Epidemiol 1996 14 1373 1379 10.1016/S0895-4356(96)00236-3 8970487

